# Human T-Cell Responses to Metallic Ion-Doped Bioactive Glasses

**DOI:** 10.3390/ijms25084501

**Published:** 2024-04-19

**Authors:** Hugo Abreu, Mari Lallukka, Marta Miola, Silvia Spriano, Enrica Vernè, Davide Raineri, Massimiliano Leigheb, Mario Ronga, Giuseppe Cappellano, Annalisa Chiocchetti

**Affiliations:** 1 Department of Health Sciences, Interdisciplinary Research Center of Autoimmune Diseases-IRCAD, Università del Piemonte Orientale, 28100 Novara, Italy; hugo.abreu@uniupo.it (H.A.); davide.raineri@med.uniupo.it (D.R.); massimiliano.leigheb@gmail.com (M.L.); mario.ronga@med.uniupo.it (M.R.); annalisa.chiocchetti@med.uniupo.it (A.C.); 2 Center for Translational Research on Autoimmune and Allergic Diseases-CAAD, Università del Piemonte Orientale, 28100 Novara, Italy; 3 Applied Science and Technology Department, Politecnico di Torino, 10129 Torino, Italy; mari.lallukka@polito.it (M.L.); marta.miola@polito.it (M.M.); silvia.spriano@polito.it (S.S.); enrica.verne@polito.it (E.V.); 4 Orthopaedics and Traumatology Unit, “Maggiore della Carità” Hospital, 28100 Novara, Italy

**Keywords:** bioactive glasses, multiparametric flow cytometry, immunobiocompatibility, metallic ion doping, tissue regeneration, inflammation

## Abstract

Biomaterials are extensively used as replacements for damaged tissue with bioactive glasses standing out as bone substitutes for their intrinsic osteogenic properties. However, biomaterial implantation has the following risks: the development of implant-associated infections and adverse immune responses. Thus, incorporating metallic ions with known antimicrobial properties can prevent infection, but should also modulate the immune response. Therefore, we selected silver, copper and tellurium as doping for bioactive glasses and evaluated the immunophenotype and cytokine profile of human T-cells cultured on top of these discs. Results showed that silver significantly decreased cell viability, copper increased the T helper (Th)-1 cell percentage while decreasing that of Th17, while tellurium did not affect either cell viability or immune response, as evaluated via multiparametric flow cytometry. Multiplex cytokines assay showed that IL-5 levels were decreased in the copper-doped discs, compared with its undoped control, while IL-10 tended to be lower in the doped glass, compared with the control (plastic) while undoped condition showed lower expression of IL-13 and increased MCP-1 and MIP-1β secretion. Overall, we hypothesized that the Th1/Th17 shift, and specific cytokine expression indicated that T-cells might cross-activate other cell types, potentially macrophages and eosinophils, in response to the scaffolds.

## 1. Introduction

Musculoskeletal disorders (MSDs) are conditions that restrict movement of the body, causing injury and pain in tissues belonging to the musculoskeletal system, including muscles, bones and joints [1,2]. Among the most prevalent MSDs are osteoarthritis, rheumatoid arthritis, low back pain and bone fractures, generally associated with osteoporosis in the elderly population [1,3]. In fact, with the increase in the average life expectancy observed in the last century, there has been a steady rise in the incidence of MSDs [2]. Individuals affected by MSDs experience a spectrum of pain and discomfort, which depending on the severity of the symptoms, can range from a slight interference with the daily activities to complete movement impairment. Therefore, there is a pressing need for novel therapies that can alleviate symptoms and improve the quality of life of MSD patients [1].

Biomaterials are designed and engineered to interact with biological systems for medical purposes. These materials play a crucial role in various fields, such as medicine, biotechnology and tissue engineering, where they can be used to replace or enhance natural biological structures or support specific functions within the body [4]. Biomaterials should be immunobiocompatible, meaning that they do not elicit a significant immune response or cytotoxicity when interacting with an organism, but also functional, whether they are functioning as a structural support, as promotors of tissue regeneration or vehicles for drug delivery. Among the different types of biomaterials on the orthopedic field, the most commonly used include the following: metals, more specifically titanium and stainless steel; polymers, either synthetic like polyethylene and polyurethane or natural such as collagen and hyaluronic acid; ceramics, for example, hydroxyapatite; bioactive glasses; and composites, which are the result of the combination of at least two different materials. Depending on their properties, these biomaterials can be used as implants, drug carriers for controlled delivery, artificial tissues/organs or diagnostic devices [5].

Damaged or diseased parts of the musculoskeletal system as well as dental abnormalities can be replaced by bioceramics since they have been modified for load-bearing purposes like bone grafts and cement, hip acetabular cups and dental implants [6,7]. Bioceramics and bioactive glasses have exceptional biocompatibility, corrosion resistance, a hard, crisp surface and osteoconductivity, i.e., the ability of bone-forming cells in the grafting area to migrate across a scaffold and gradually replace it with new bone tissue over time. Furthermore, they can directly interact with the living surrounding tissue and show convincing effects on wound healing after implantation, as in the cases of bioactive glasses and hydroxyapatite (HA). Due to their low friability, they are usually used in dental abnormalities and small bone fillings [7].

Among the described materials, bioactive glasses represent an interesting option due to their excellent biocompatibility and bioactivity. They are able to form a bond with mineralized bone tissue in the physiological body environment by creating a calcium phosphate layer on their surface [8]. Over fifty years ago, Larry Hench introduced bioactive glasses, more specifically Bioglass^®^ 45S5, the first commercially available glass for medical use [9,10]. The composition of most bioactive glasses is based on silica, sodium oxide, calcium oxide and phosphorous pentoxide. This composition allows for the alteration or combination of these basic elements, enabling the creation of different types of bioactive glasses with specific properties such as bone forming efficiency, degradability, antibacterial properties and even soft tissue regeneration and wound healing [11,12,13]. When the bioactive glass is implanted, it releases its main ions (calcium, sodium, phosphate and silica) to form carbonated hydroxyapatite (HCA), a bone-like mineral coating, through an ion exchange reaction between the glass surface and the surrounding tissue and fluids [13]. This apatite layer improves cellular adhesion and proliferation of osteogenic cells and it is gradually replaced by bone over time [14,15]. Conversely, excessive ion release may lead to undesired toxicity, therefore affecting cell viability and metabolic activity, which in turn can impair the tissue healing process. To counteract this effect, pre-treating the bioactive glasses prior to entering into contact with cells by incubating the material in cell culture medium or buffer can be a successful strategy [16].

In summary, advancements in biomaterials science have led to the development of increasingly sophisticated materials with tailored properties, enabling innovative solutions in healthcare and biotechnology. Researchers continually explore new biomaterials and their applications to improve patient outcomes and quality of life. However, in a living tissue, when a material is implanted, there will always be a physiologic immune response which represents the first step of tissue repair. Nowadays, biomaterials are being designed considering this immune response and modulating it for improving implant integration, avoiding the chronic inflammatory and foreign body reactions that may lead to the loss of function [17,18].

Fibroblasts and macrophages have been traditionally used for evaluating biomaterial compatibility, such as in the case of silicone breast implants [19]. In the specific case of musculoskeletal regeneration, cytotoxicity is commonly evaluated on mesenchymal stem/stromal cells (MSCs) and osteoblasts [20], while the role of T-cells in tissue regeneration has only recently been explored; as of today, the reliable data on the role of T-cells are scarce [19,21]. The activation of the immune response requires three signals. Signal 1 is mediated by the binding of the T-cell receptor (TCR) to major histocompatibility complex (MHC) class molecules on antigen-presenting cells (APC); signal 2 is mediated by the engagement of co-stimulatory molecules such as B7.1 (CD80) and B7.2 (CD86) and lastly, cytokines drive the polarization of differentiated T helper (Th) cells towards several subsets, such as T helper (Th)-1, Th2, and to a lesser extent Th17 (signal 3) [22].

T-cells represent up to 70% of peripheral blood mononuclear cells (PBMCs) [23] and are able to modulate bone healing and osteogenesis through cytokine and growth factors secretion [21]. In fact, in vivo experiments showed that T-cell depletion directly impairs the osteoinduction process [24], particularly affecting the deposition of collagen and osteoblast organization [25]. In vitro, conditioned media of CD4^+^ Th lymphocytes can promote mesenchymal stem/stromal cell (MSC) mineralization [26].

Overall, the aim of this work was to elucidate how the interaction of T-cells with several metal-doped silica-based bioactive glasses, more specifically silver, copper and tellurium, could affect cell viability, T-cell immunophenotype and cytokine secretion. Silver and copper were introduced in the bioactive composition using the ion-exchange process, while tellurium was inserted together with the starting reactants during the glass synthesis via the melt and quenching process. These elements were selected since they have a therapeutic effect; Ag, Cu and Te possess antibacterial properties [27,28,29], Ag and Cu also have a pro-angiogenic effect [30,31] and Te possesses antioxidant properties [32]. However, the amount must be carefully tailored to avoid cytotoxic effects.

## 2. Results

Extensive physicochemical surface characterization of the doped glasses were previously reported by the authors [27,32,33,34,35]. Table 1 compares the most relevant properties of the glasses, regarding the content of the doped metal obtained via energy-dispersive X-ray spectroscopy (EDS) analysis, and the amount of doped metal ion leaching both in simulated body fluid (SBF) and in a cell medium obtained via inductively coupled plasma (ICP) spectroscopy, either combined via optical emission spectroscopy (OES) or mass spectrometry (MS).

### 2.1. Silver, but Not Copper or Tellurium Ion Doping Induces Apoptosis of PBMCs

**Figure 1:**
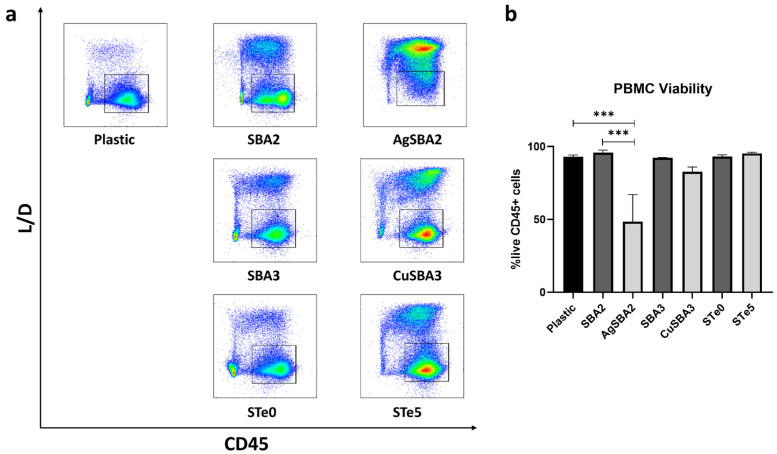
PBMC viability assay via flow cytometry. (**a**) Gating strategy: Viable lymphocytes were gated as negative for BD Horizon™ Fixable Viability Stain 780 (L/D^−^) and CD45^+^. (**b**) Bar graph representation of flow cytometry results are shown as average ± SEM. (n = 4). Ordinary one-way ANOVA with Tukey’s post-hoc correction test was used. *** *p* < 0.001.

Ions released from biomaterials upon contact with cell culture media may exert a toxic effect on immune cells. We evaluated the viability of PBMCs via flow cytometry after 48 h of culture, as seen in Figure 1a, using a fixable dye that can only be internalized by cells with permeable membranes, indicative of dead cells. In fact, we observed a reduction of approximately 50%, on average, of PBMC viability when in contact with the silver-doped bioactive glass (Figure 1b), therefore, this formulation was excluded from the further analysis. No significant differences were observed between the formulations containing copper and tellurium, along with their corresponding controls, when compared to the basal condition lacking any bioactive glass disc.

### 2.2. Immunophenotyping Reveals Th1/Th17 Shift Linked with Copper but Not Tellurium-Doping

**Figure 2:**
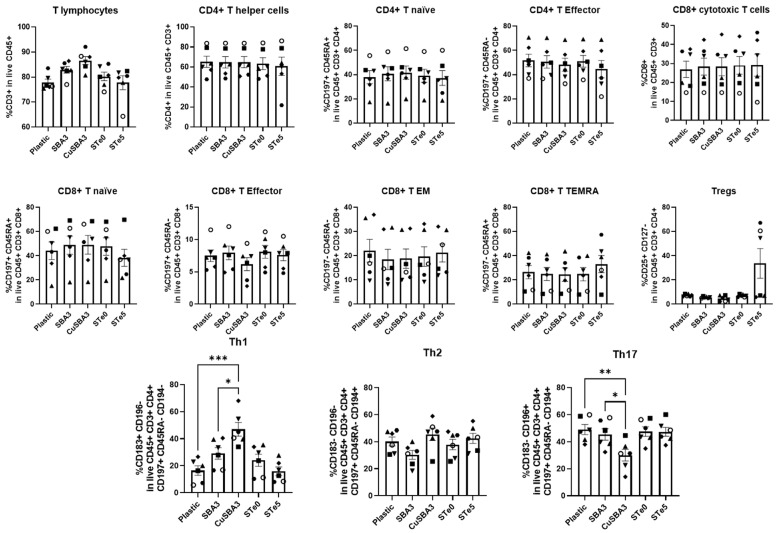
Immunophenotype of T-cells cultured in contact with bioactive glass discs assessed via multiparametric flow cytometry. Graphs represent the percentages of immune cells after 48 h culture without biomaterial (cell culture plate plastic—control), in contact with copper-doped (CuSBA3) and tellurium-enriched discs (STe5) and their respective undoped controls (SBA3 and STe0, respectively). Data are shown as average ± SEM, (n = 6). Each symbol represents a different donor. According to the data normality (Shapiro–Wilk test), ordinary one-way ANOVA (with Tukey’s post-hoc correction) or Kruskal–Wallis test (with Dunn’s post-hoc correction) were used. * *p* < 0.05, ** *p* < 0.01, *** *p* < 0.001.

To assess the immune response to the metal ion doping, PBMCs were cultured on top of bioactive glasses. In particular, the immunophenotype of T lymphocytes was evaluated via multiparametric flow cytometry, as depicted in Figure 2. The employed gating strategy (Supplementary Figure S1) allowed for the detection of several subsets of T lymphocytes, namely CD4^+^ T helper (Th) and CD8^+^ cytotoxic T-cells, further specified as naïve, effector, effector memory (EM) or terminally differentiated EM (TEMRA), based on the expression of CD45RA and CD197, regulatory T-cells (Tregs), CD25^+^CD127^−^, as well as Th1, Th2 and Th17, identified by their differential expression of CD183, CD194 and CD196 markers.

Results showed that T-cells cultured in contact with copper-doped bioactive glass discs exhibited an increased frequency of Th1 population, paralleled by a decrease in Th17 cells, compared with the negative control and to a lesser extent with its undoped counterpart. On the other hand, the tellurium-doped glass did not exhibit any statistically significant differences, although large standard deviations were verified in several subsets, more noticeably in the Treg population.

### 2.3. Cytokine Profile Response to Ion-Doped Bioactive Glasses

**Figure 3:**
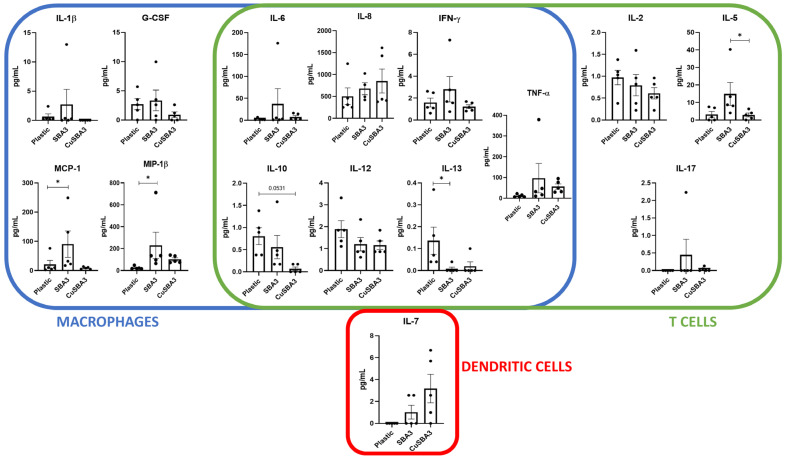
Cytokine expression levels of supernatants of PBMC culture in contact with copper-doped bioactive glass discs (CuSBA3) and its respective control (SBA3). Data are shown as average ± SEM, (n = 5). According to the data normality (Shapiro–Wilk test), RM one-way ANOVA (with Bonferroni post-hoc correction) or the Friedman test (with Dunn’s post-hoc correction) were used. * *p* < 0.05.

Considering the phenotypical variances observed during PBMC culture related exclusively to the copper-doped glass, in order to verify if the Th1/Th17 shift was paralleled by differential cytokine secretion, a further cytokine quantification ELISA was performed using a commercial multiplex kit. This comprised seventeen cytokines released from macrophage or T-cells exclusively, or by both cell populations (Figure 3). Among these, levels of GM-CSF and IL-4 were close to, or below the lower limit of quantification and thus, were not suitable for statistical comparison.

We found a significantly reduced concentration of IL-5 in the supernatant of PBMCs cultured in contact with the copper-doped glasses condition, compared with its undoped counterpart. Copper-doped glasses also showed a trend in the decrease in IL-10 cytokine secretion in comparison with the control (plastic). Furthermore, IL-13 levels were significantly decreased in the undoped condition in comparison with plastic, while MCP-1 and MIP-1β cytokines were significantly increased. These findings indicated that the copper-doped bioactive glass exhibited a more similar cytokine profile to the plastic rather than to its respective undoped bioactive glass discs.

## 3. Discussion

Bioactive glasses commonly release their ions to the surrounding microenvironment. This opens the possibility of incorporating biologically active ions into their composition or onto their surface, which upon release, may promote specific biological functions such as cell proliferation or angiogenesis, or confer novel properties to the bioactive glass, for example an antimicrobial effect [36]. Thus, silver, copper and tellurium have been used as doping ions on the surface of bioactive glass discs. For Ag- and Cu-doped glass, the ion doping was performed via ion exchange which introduced the ions only on to the glass surface, while in the case of STe5, tellurium was part of the bulk glass network. These ions have already been studied in the context of their antimicrobial [27,28,29] and pro-angiogenic effects [30,31], as well as in bone tissue regeneration and cancer [37,38,39]. Nevertheless, the data regarding the impact of these formulations on T-cells are scarce. Considering this, and to extend the knowledge provided by previous studies on biofilm formation [27,28,32], we evaluated any effects on viability, T-cell immunophenotype and cytokine release of PBMCs cultured in contact with ion-doped bioactive glasses.

The ionic release test was performed using inductively coupled plasma-optical emission spectrometry (ICP-OES) and data on doped ion-leaching in both cell culture medium and simulated body fluid was added to Table 1. Regarding the doping ions’ bio-assimilation, it was reported that blood cells like erythrocytes and macrophages are capable of assimilating Cu^2+^ through copper transporter 1 (CTR1) [40]. Also, silver ions (Ag^+^) seem to be captured by immune cells, such as neutrophils and macrophages, and exert similar effects as silver nanoparticles, specifically in the formation of neutrophils extracellular traps (NETs) and intracellular reactive oxygen species (ROS) [41]. Lastly, tellurite (TeO_3_^2−^) which releases Te^4+^ and represents the most abundant form of tellurium in nature, has been shown not only to bind hemoglobin in erythrocytes but also to react with glutathione and to lead to ROS formation in leukocytes [42]. The cytotoxicity of the released ions (Ag^+^, Cu^2+^, Te^4+^) on different cell lines was investigated in previous reports [27,28,29,30,31], showing a different behavior based on the used method (indirect or direct). Any observed cytotoxic effects, in particular for copper, do not seem to arise from the dissolution products or specific ion concentrations in the medium, but rather from a burst release and contact toxicity with the doped glass surfaces. A recent publication by some of the coauthors used copper-doped bioactive glasses manufactured with the same methodology and evaluated its cytotoxicity using human adipose tissue-derived stem cells (hASCs) [33]. Indirect culture of hASCs with the conditioned media of CuSBA3 discs, soaked for 24 h in α-MEM supplemented with 5% human serum and 1% antibiotics (100 U/mL penicillin and 0.1 mg/mL streptomycin), did not affect cell viability, while direct contact with CuSBA3 led to extreme cytotoxicity. In accordance, when fibronectin was incorporated onto the surface of CuSBA3, ASCs cytocompatibility remained low, since the coating provided support for cell attachment but it did not prevent the direct contact between cells and bioactive glass disc. On the other hand, allowing the excessive burst ion release prior to the cell seeding through a 24 h pre-incubation in α-MEM rendered CuSBA3 cytocompatible [33]. In our study, we tested PBMCs directly cultured on the top of bioactive glasses. These cells mostly comprised non-adherent cells, therefore they tended not to be in direct contact with the bioactive glass discs. Given that, our data on PBMC viability are in line with the prior report regarding the indirect assays performed with adherent hASCs [33], in which the concentration of ions in the solution, about 10,000 μg/L, did not significantly impact cell viability. Overall, the reported cytotoxicity of copper-doped bioactive glasses was likely due to the contact but not necessarily to the concentration in solution.

In our study, we report the highly toxic effect of silver on PBMCs, as demonstrated by significantly reduced viability of PBMCs. Previous studies showed that the elementary silver in the solution, not doped in any biomaterial, exhibits cytotoxicity in a dose-dependent manner [43]. While generally considered an element with low toxicity [44], there are some clinical data indicating that the exposure to silver may represent the primary cause responsible for damage in cornea, liver, kidney and neurological tissues [45], as well as causing leukopenia [46] and chronic heart inflammation [47]. Moreover, in vitro studies presented some drawbacks of its use, due to a notable impairment of fibroblast [48] and keratinocyte growth [49]. For the above reasons, we opted to focus our attention on the copper- and tellurium-doped formulations for further analyses.

Delving into the T-cell ion-induced phenotype, the main focus of our study, we found that both the tellurium-doped bioactive glass and its undoped control exhibited no discernible impact on the immunophenotype of T-cells. However, we did observe interindividual variability, commonly present when using primary cells, particularly evident in certain subsets, as shown by the high standard deviations detected. In this case, PBMC culture in contact with the tellurium-doped bioactive glass revealed that three out of six donors had a high frequency of Tregs (54.5–67.1%) while the other three donors exhibited a much lower frequency (5.8–7.1%). Also in other subsets, such as CD4^+^ T helper and CD8^+^ T TEMRA, albeit less noticeable, we still verified that the culture with the tellurium-doped bioactive glass caused a more variable effect on PBMC phenotype than the other conditions, which might indicate that the response to this element was highly subject-dependent. Although material implantation generally elicits a response by the host, recent biomaterial engineering approaches search to not only modulate it in order to minimize side effects, such as chronic inflammation and foreign body reaction, but also to attempt to improve desirable biological processes, for instance osteointegration [18,50]. Previous reports evidencing the protective effect of the tellurium doping against oxidative stress coupled with our findings reporting no significant alterations in T-cell phenotype, can indicate that the tellurium-doped bioactive glass can be an interesting alternative to the currently used biomaterials for implantation [32].

On the other hand, culturing PBMCs in contact with the copper-doped bioactive glass led to a significant increase in Th1 cells, accompanied by a decrease in Th17 cells, compared with both its undoped counterpart (ordinary one-way ANOVA with Tukey’s post-hoc correction; Th1: SBA3 vs. CuSBA3, *p*-value = 0.0332; Th17: SBA3 vs. CuSBA3, *p*-value = 0.0498) and plastic (ordinary one-way ANOVA with Tukey’s post-hoc correction; Th1: CuSBA3 vs. Plastic, *p*-value = 0.0001; Th17: CuSBA3 vs. Plastic, *p*-value = 0.0097). Both cell subsets were derived from the polarization of naïve T-cells, typically exacerbated upon viral and bacterial infections, inducing cell-mediated immunity, mainly by stimulating antibody secretion from B-cells. In addition, both Th1 and Th17 cells are known for their pro-inflammatory phenotype due to their effector cytokine releases. However, their biogenesis and role in the immune system are different. While Th1 cells are generated in the presence of IL-12, IL-18 and IFN-γ cytokines, Th17 requires IL-6, IL-23 and TGF-β for its polarization. Furthermore, Th1 mainly produce IFN-γ and TNF-α, while Th17 cells generally release IL-17A, IL-17F and IL-22. Consequently, their functions are also distinguishable. Th1 are able to enhance APC activity and CD8^+^ T-cells/macrophage activation, protect against intracellular pathogens and participate in delayed type hypersensitivity, while Th17 acts on fungal and extracellular bacterial infections. Interestingly, Th subsets can cross-regulate each other, meaning that the secreted products of one cell type can stimulate the polarization of CD4^+^ naïve cells into another specific subset. Finally, Th cells are known to be highly plastic; Th17 cells are considered less stable and can for example differentiate into Th1 cells given the appropriate environmental setup [22]. As reviewed by Adusei et al., Th subsets are capable of modulating the processes of fibrosis and tissue regeneration through differential cytokine secretion [51]. T-cells were studied on fibrotic tissue developed upon silicone implantation where an increase in CD4^+^ T-cells was detected in the capsular tissue, more notably, Tregs. Moreover, Tregs were more prominent in patients with milder symptoms compared to more severe cases, showing also a more suppressive effect in vitro. Additionally, the authors showed an increase secretion of IL-6, IL-8, IL-17, IFN-γ and TGF-β1 by immune cells present in the capsular tissue, indicative of a pro-inflammatory environment sustained by Th1 and Th17 cells [52].

The direct correlation between copper-doping in bioactive glasses and Th subsets is still ill-defined. However, understanding the complex interactions between the different constituents of the immune system can help elucidating this question. Professional APCs, such as dendritic cells (DCs) and macrophages, are responsible for T-cell activation, therefore any modulatory effect on these players can influence the state of Th cells [22]. Dey et al. reported that the addition of copper oxide nanoparticles to lymphocytes or macrophages in vitro leads to an increase in TNF-α, IFN-γ and IL-12 production; the latter two directly promote the polarization of CD4^+^ T-cells towards the Th1 phenotype [53]. Although we have not detected any significant changes regarding the aforementioned cytokines, it should be noted that we have evaluated the cytokine levels at 48 h instead of the 24 h used by the authors. Additionally, the physicochemical properties of nanoparticles also differ from the bulk materials, which may lead to slightly diverse biological reactions [54]. Nevertheless, the increase in the percentage of Th1 cells upon culture in contact with the copper-doped bioactive glass in our setting might indicate a similar pro-inflammatory effect of the copper doping.

Schuhladen et al. have evaluated the effect of increasing concentrations of copper-doped bioactive glass nanoparticles (Cu-BG-NPs) on murine DCs’ phenotype and function. The authors found that the conditioned media containing the ionic dissolution products of Cu-BG-NPs significantly reduced the expression of CD80 and CD86, the two ligands of CD28, which are essential for T-cell activation. Coupling the phenotype results with the cytokine expression evaluation, the authors concluded that higher concentrations of copper led to a decrease in the secretion of various cytokines such as IL-6 [55]. As previously mentioned, IL-6 is one key factor for the polarization of Th17 cells, therefore its copper-induced reduction might lead to a lower frequency of Th17 cells, which is in line with our results.

Interestingly, the cytokine evaluation of supernatants from PBMC culture with the copper-doped bioactive glass revealed a distinct pattern, different to what had been previously described. IL-5 was revealed as the only cytokine being significantly modulated between the copper-doped and its undoped control. Within the immune system, IL-5 is commonly produced by Th2, innate lymphoid cells type 2 (ILC2), mast cells, natural killer cells and eosinophils, acting particularly on eosinophil and B-cell growth [56,57]. In our setting, this cytokine appeared to be downmodulated in the copper-doped bioactive glass, achieving values similar to the negative control. Strikingly, another member of its family typically associated with Th2 response [58], namely IL-13, showed an inverse pattern, being under expressed in the undoped control in comparison with both other conditions. Notably, the proportion of Th2 cells did not change according to our immunophenotype results, even though their biological processes could be differentially modulated, therefore resulting in diverse secreted products.

Although often associated, even potentially acting upon some of the same molecules such as the signal transducer and activator of transcription 6 (STAT6), IL-5 and IL-13 are markedly different. As reviewed by Wu et al., IL-5 is mostly responsible for eosinophils’ biological processes and survival, while IL-13 impacts more directly the B-cells and the Th2 subset [59]. Comparing with our results, the overexpression of IL-5 accompanied by a downregulation of IL-13 might lead to IL-5-mediated eosinophil activation, produced by Th2 cells. Therefore, it would be relevant to clarify through in vivo testing the role of eosinophils in this context, considering that the tested PBMC fraction should contain only a residual percentage of these cells. In fact, the formation of eosinophilic clusters had been reported in mice after six weeks of bioactive glass implantation, which the authors considered a sign of a possible allergic reaction [60]. Overall, we consider that in the SBA3 condition, the IL-5 likely produced by Th2 cells will or could favor eosinophil activation instead of B-cells, due to the lack of IL-13.

Conversely, two chemokines were upregulated in the undoped bioactive glass, more specifically MCP-1/CCL2 and MIP-1β/CCL4, which are responsible for immune cell recruitment. Both are mostly produced by cells from the myeloid lineage, i.e., monocytes, macrophages and dendritic cells, although they can also be released by T-cells [61,62]. These cytokines have been already linked with the immune response to biomaterials, being secreted by neutrophils [63], macrophages [64] and T-cells [65] (reviewed in [18]). The fact that these pro-inflammatory chemoattractant molecules were significantly more present in the undoped condition might favor macrophage polarization into M1 phenotype, which can be responsible for balancing the inflammatory microenvironment [66], acting as a counterpart of the activated eosinophils.

Of note, no significant differences were found at the cytokine level between the copper-doped condition and the basal condition, without any bioactive glass, although in the case of IL-10, a trend of reduced production upon PBMC culture with the copper-doped discs was observed. The impact of this cytokine in fibrosis seems a paradox. While it is commonly linked to a type 2 response [66], it may also play a role in preventing or reducing the effects of fibrosis [67], therefore further research on this topic is needed (reviewed in [68]).

Our study presents some limitations. Macrophages are among the first cell types to interact with implants, therefore being the main focus of numerous studies that explore the impact of implants in the immune system. The multiplex ELISA panel used in this study allowed for an overview of the cytokines released by PBMCs due to the contact with the bioactive glasses, it did not permit the direct association of the cytokine profile with a specific subset, such as macrophages. Nevertheless, among the cytokines evaluated in PBMC cultures, two were mainly ascribed to macrophages, such as MCP-1 and MIP-1β. Even though the literature already describes the modulation of cytokine release in macrophages by metal ion-doped bioactive glasses, including copper [69,70,71,72,73], it would be of interest to complete the analysis of T-cell immunophenotyping with their interaction with macrophages by polarizing them in vitro. Second, we had to take into account our sample size and the observed variability of donors in their response to the different biomaterial. This variability reflected the diverse genetic backgrounds, immune statuses, and physiological conditions of individual donors that may affect the response. Third, our experimental design investigated the effects of each biomaterial in a static way, though in vivo, these biomaterials are aimed at repairing and reconstructing the defective bone which is a dynamic tissue and subjected to mechanical stress. Lastly, the tissue microenvironment could also induce the release of ions from each biomaterial by affecting the T-cell/macrophages interaction, even by modulating macrophage polarization, shifting them towards an anti-inflammatory profile [74].

Overall, the fact that the cytokine profile of the copper-doped bioactive glass was similar to the plastic control, together with the favoring of the Th1 response according to the immunophenotyping assay, indicates that copper-doping might be a valid strategy to prevent fibrotic tissue formation.

## 4. Materials and Methods

### 4.1. Bioactive Glasses Preparation

In the present study, silica-based bioactive glasses were used as bulk discs. These materials were prepared and characterized as previously reported [27,32,33,34,35]. Briefly, the composition of SBA2 and SBA3 (undoped controls) are shown in Table 2.

SBA2 and SBA3 were prepared via the melt and quenching process. The reactants were melted in a platinum crucible at 1450 °C for 1 h Subsequently, the melt was cooled in a brass mold to obtain glass bars, which were then annealed at 500 °C for 13 h and cut into slices of 2 mm thickness and about 1 cm in diameter. Then, the slices were polished with SiC abrasive papers up to 1200 grit to level the surfaces. Lastly, the introduction of silver (Ag^+^) and copper (Cu^2+^) ions onto the surface of SBA2 and SBA3, respectively, was achieved through the ion-exchange process. The discs were submerged in an aqueous solution of either AgNO_3_ (30 mM) or Cu(CH_3_COO)_2_ (1 mM) for 1 h, at 37 °C.

In the case of Te-doped glass (STe5), tellurium was directly introduced into the composition of the bioactive glass (named STe0) as a substitute for silica, as reported in Table 3.

STe0 and STe5 were also prepared via the melt and quenching process. In this case, the reactants were melted in a platinum crucible at 1500 °C for 1 h, and then cooled in a brass mold to obtain glass bars, that were annealed at 550 °C for 13 h. These bars were cut into slices of similar dimensions as the previous discs and polished as before. All the aforementioned samples were sterilized by heating to 100 °C for 3 h.

### 4.2. Ion Release in Simulated Body Fluid (SBF) and Cell Medium

The ICP analyses summarized in Table 1, except for STe5, were previously published and reported [33,35]. The glass samples of STe5 were subjected to in vitro bioactivity tests by soaking them in simulated body fluid (SBF). The SBF was prepared using the protocol developed by Kokubo et al. [75]. Polished glass discs were immersed in 50 mL of SBF for fixed periods (1, 3, 7, 14, 28 days, here reported only 3-day timepoint) with five replicate samples of each glass per time point. Samples were maintained at 37 °C in an incubating shaker with an orbital speed of 120 rpm to simulate the physiological fluid flow. Solution at each time point was collected and the cumulative ion release for each sample was calculated by adding the ion release value at the selected time point to the previous ones. In the case of Te-ion release in cell medium (DMEM high glucose medium (Euroclone, Pero, Italy), supplemented additionally by penicillin/streptomycin and L-Glutamine (1% of both), STe5 specimens were soaked in the medium without FBS, 1.5 mL per disk in triplicates. Samples were maintained in the solution for 3 days, at 37 °C, 5% CO_2_ incubation. From both SBF and cell medium collected samples, the Te-ion release was determined via an inductively coupled plasma mass spectrometer (ICP-MS, iCAPTM Q, Thermo Fisher Scientific, Waltham, MA, USA).

### 4.3. Blood Specimen Collection

Peripheral blood was obtained from six healthy adult donors (25–45 years old) in cooperation with the Hospital Maggiore della Carità, Novara, Italy. From each donor, 10 mL of peripheral blood was withdrawn into lithium heparin collection tubes and immediately processed. The study was approved by the local ethics committee (prot. n. 675/CE).

### 4.4. Peripheral Blood Mononuclear Cells (PBMCs) Isolation

PBMCs were isolated from heparinized blood samples collected from healthy donors. The blood samples were mixed with equal amounts of phosphate buffer saline (PBS 1×) and were carefully overlaid on top of a density gradient isolation solution, Lympholyte-H (Cedarlane^®^, Burlington, ON, Canada). After centrifugation, the cell ring at the interface was collected, washed with PBS 1X, and cells were counted.

### 4.5. Assessment of Cell Viability via Flow Cytometry

PBMCs from four healthy adult donors were cultured in RPMI 1640, supplemented with 10% (*v*/*v*) heat-inactivated FBS, 100 U/mL penicillin/streptomycin, and 100 μg/mL gentamicin (Life technologies, Carlsbad, CA, USA), at 37 °C and 5% CO_2_. A total of 1 × 10^6^ fresh cells/mL were seeded onto sterile discs of bioactive glasses (or in wells without bioactive glasses—negative control) for 48 h, keeping the polished side upwards. Afterwards, the media was removed, and cells were collected and washed with PBS-EDTA 2 mM. The cells were stained with a viability dye (BD Horizon™ Fixable Viability Stain 780 (Becton and Dickinson, Franklin Lakes, NJ, USA)) for 15 min, at 4 °C, to distinguish live and dead cells. After washing with PBS-EDTA, Human BD™ Fc block solution was added to block the non-specific binding of immunoglobulin to Fc receptors. Subsequently, cells were incubated with antiCD45 BUV395 mAb (clone: HI30), a pan-marker for all leukocytes. Lastly, the cells were washed and resuspended in PBS-EDTA for acquisition using a BD FACSymphony™ A5 flow cytometer (BD Biosciences, Franklin Lakes, NJ, USA). The samples were then analyzed using the BD FACSDIVA™ software version 9.0.

### 4.6. Immunobiocompatibility Assay

PBMCs from six healthy adult donors were cultured as previously described. After media removal, cells were washed with PBS-EDTA 2 mM and stained with a viability dye (BD Horizon™ Fixable Viability Stain 780) for 15 min, at 4 °C. Cells were then washed with PBS-EDTA and Human BD™ Fc block solution was added. Antigen surface staining was performed by adding an antibody mix containing mouse antiCD3 BUV496 monoclonal antibody (mAb) (clone: UCHT1), antiCD4 BUV737 mAb (clone: SK3), antiCD8 BUV805 mAb (clone: SK1), antiCD25 APC-R700 mAb (clone: 2A3), antiCD45 BUV395 mAb (clone: HI30), antiCD127 BV786 mAb (clone: HIL-7R-M21), antiCD45RA BUV563 mAb (clone: HI100), antiCD183 APC mAb (clone: IC6), antiCD194 PE-CF594 mAb (clone: 1G1), antiCD196 BV480 mAb (clone: 11A9) and antiCD197 BV711 mAb (clone: 150503) in BD Horizon™ Brilliant Stain Buffer for 20 min, at 4 °C. Lastly, the cells were washed and resuspended in PBS-EDTA for acquisition using a BD FACSymphony™ A5 flow cytometer. Data were then analyzed using the BD FACSDIVA™ software version 9.0. All reagents were purchased from Becton and Dickinson (Franklin Lakes, NJ, USA).

### 4.7. Enzyme-Linked Immunosorbent Assay (ELISA)

PBMCs from five healthy adult donors were cultured as previously described. Cell culture supernatants were collected after 48 h and cytokine levels were quantified using the Bio-Plex Pro Human Cytokine 17-plex Assay according to manufacturer’s instructions (Bio-Rad, Hercules, CA, USA). This assay allows for the detection of a wide array of cytokines, specifically: G-CSF, GM-CSF, IFN-γ, IL-1β, IL-2, IL-4, IL-5, IL-6, IL-7, IL-8, IL-10, IL-12, IL-13, IL-17A, MCP-1, MIP-1β and TNF-α. The plate was run on a Bio-Plex 200 instrument (Bio-Rad, Hercules, CA, USA). The reported concentrations and detection limits were obtained through the standard curves generated by the kit’s standards, using the weighted 5PL curve fitting procedure in Bio-Plex Software Manager ™ version 6.2. Values under the lower limit of quantification (LLOQ) were extrapolated based on the 5PL logistic curve, as previously reported [76].

### 4.8. Statistical Analysis

Data were analyzed using one-way ANOVA, Friedman or Kruskal–Wallis test with post-hoc correction, according to the sample’s normality, calculated using the D’Agostino–Pearson test. *p*-value below 0.05 was considered statistically significant. Statistical analyses were performed with GraphPad Instat software (Prism 8 version 8.4.3) (GraphPad Software, San Diego, CA, USA).

## 5. Conclusions

Although bioactive glasses have been used especially for hard tissue regeneration during the last decades, the complete evaluation of immune reaction towards these biomaterials is often lacking. Our approach targeted T-cells that can be responsible for many regulatory functions in the organism including inflammation, which is essential for tissue regeneration. Our findings showed not only that metal ion doping can cause the apoptosis of immune cells and modulate the expression of certain subsets of T-cells in vitro, but it can also impact the cytokine release. Our study reported the highly toxic effect of silver-doping on PBMCs, comparable to the known dose-dependent cytotoxicity of this element in solution, indicating that this formulation required further optimization before being used in in vivo studies. Even though tellurium-enriched bioactive glass did not notably affect PBMC viability, the presence of tellurium elicited a highly variable T-cell response among individuals, most notably within the Treg subset. Additional research is necessary to investigate the distinct immune responses of each individual to the presence of this ion. In regards to the copper-doping, we postulated that the Th17 to Th1 switch, together with the alteration in cytokines such as IL-5 and IL-13 and the chemokines MCP-1/CCL2 and MIP-1β/CCL4, can modulate the immune response to bioactive glass implantation through cross-activation of cell types other than T lymphocytes, such as macrophages and possibly eosinophils. More importantly, the incorporation of copper on the surface of the bioactive glass greatly brought back the cytokine expression to the basal condition without biomaterial by improving its immunobiocompatibility.

Further studies are also needed to evaluate the effect of copper-doped bioactive glass in in vivo settings, where all the relevant players in the inflammatory response associated with tissue regeneration are present.

## Figures and Tables

**Table 1:** Summary of the properties of the doped glasses [33,35]; * α-MEM without added serum, 5% antibiotics, ** DMEM High Glucose, 1% L-Glutamine, 1% antibiotics. None of the used media contained any cells.

	AgSBA2	CuSBA3	STe5
Doping method	The surface of the glass (Ion exchange in aqueous solution of AgNO_3_)	The surface of the glass (Ion exchange in aqueous solution of Cu (CO_2_CH_3_)_2_)	The bulk of the glass (TeO_2_ in the glass network as an oxide)
Doped element content at-% (EDS)	0.7 ± 0.36	8.4 ± 0.18	3.4 ± 0.08
Doped ion leaching after 3 days in cell medium	7.9 ± 1.4 ppm * (ICP-OES)	11.0 ± 2.4 ppm * (ICP-OES)	5.6 ± 0.3 ppm ** (ICP-OES)
Doped ion leaching after 3 days in Simulated Body Fluid (SBF)	0.37 ± 0.13 ppm (ICP-MS)	0.14 ± 0.04 ppm (ICP-MS)	0.21 ± 0.07 ppm (ICP-MS)

**Table 2:** Composition of SBA2 and SBA3 control bioactive glasses.

Components	SBA2 (% mol)	SBA3 (% mol)
SiO_2_	48	48
Na_2_O	18	26
CaO	30	22
P_2_O_5_	3	3
B_2_O_3_	0.43	0.43
Al_2_O_3_	0.57	0.57

**Table 3:** Composition of the STe0 control bioactive glass and STe5-doped bioactive glass.

Components	STe0 (% mol)	STe5 (% mol)
SiO_2_	48.6	43.6
Na_2_O	16.7	16.7
CaO	34.2	34.2
P_2_O_5_	0.5	0.5
TeO_2_	0.0	5.0

## Data Availability

The authors declare that the data supporting the findings of this study are included within the article or Supplementary Materials and are available from the corresponding author upon reasonable request.

## Supplementary Materials

The following supporting information can be downloaded at: https://www.mdpi.com/article/10.3390/ijms25084501/s1.
