# Supplementary material for: Human T-Cell Responses to Metallic Ion-Doped Bioactive Glasses
Source: Int J Mol Sci. 2024 Apr 19;25(8):4501. doi: 10.3390/ijms25084501 (PMC11050560; doi:10.3390/ijms25084501)
Supplement: Supplementary file 1 [file ijms-25-04501-s001.zip › ijms-2942596-supplementary.pdf]

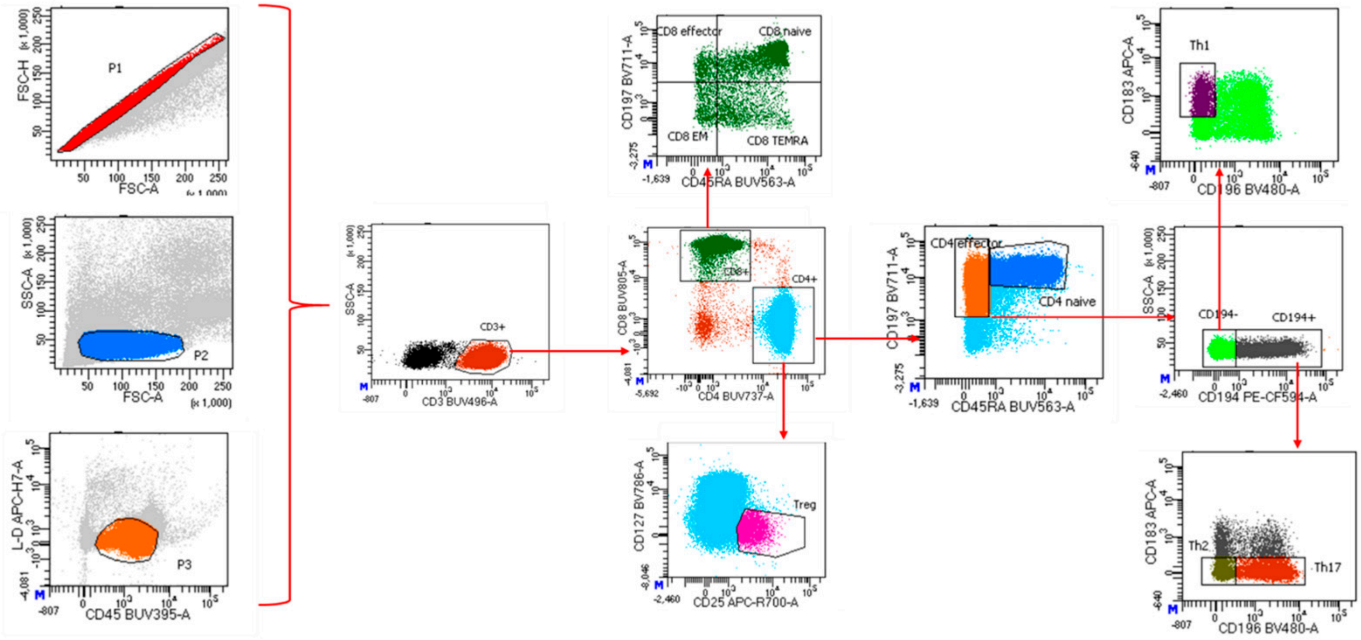

Figure S1. Gating strategy. Arrows describe the hierarchical sequences of analysis. Singlets were selected from the FSC-A versus FSC-H dot plot and cells were gated based on SSC-A versus FSC-A. Live lymphocytes were gated as CD45^{+} and Fixable Viability Dye 780^{-} (L/D^{-}). T cells were identified as CD3^{+}. CD4^{+} and CD8^{+} T cells were identified accordingly to the expression of their surface markers. In the CD8 subset, naïve cells were classified as CD197^{+}CD45RA^{+}, effector cells as CD197^{+}CD45RA^{-}, effector memory cells as CD197^{-}CD45RA^{-}, and terminally differentiated effector memory cells as CD197^{-}CD45RA^{+}. In the CD4 subset, naïve cells were classified as CD197^{+}CD45RA^{+}, and effector cells as CD197^{+}CD45RA^{-}. Tregs cells were classified as CD4^{+}CD127^{-}CD25^{+}. T helper 1 cells were classified as CD194^{-}CD183^{+}CD196^{-}, T helper 2 as CD194^{+}CD183^{-}CD196^{-} and T helper 17 as CD194^{+}CD183^{-}CD196^{+}.
